# Drivers of Phosphorus Uptake by Barley Following Secondary Resource Application

**DOI:** 10.3389/fnut.2016.00012

**Published:** 2016-05-12

**Authors:** Eva Brod, Anne Falk Øgaard, Tore Krogstad, Trond Knapp Haraldsen, Emmanuel Frossard, Astrid Oberson

**Affiliations:** ^1^NIBIO, Norwegian Institute of Bioeconomy Research, Ås, Norway; ^2^Department of Environmental Sciences, Norwegian University of Life Sciences, Ås, Norway; ^3^Institute for Agricultural Sciences, ETH Zürich, Lindau, Switzerland

**Keywords:** waste products, fish sludge, meat bone meal, wood ash, animal manure, isotope techniques

## Abstract

Minable rock phosphate is a finite resource. Replacing mineral phosphorus (P) fertilizer with P-rich secondary resources is one way to manage P more efficiently, but the importance of physicochemical and microbial soil processes induced by secondary resources for plant P uptake is still poorly understood. Using radioactive-labeling techniques, the fertilization effects of dairy manure, fish sludge, meat bone meal, and wood ash were studied as P uptake by barley after 44 days and compared with those of water-soluble mineral P (MinP) and an unfertilized control (NoP) in a pot experiment with an agricultural soil containing little available P at two soil pH levels, approximately pH 5.3 (unlimed soil) and pH 6.2 (limed soil). In a parallel incubation experiment, the effects of the secondary resources on physicochemical and microbial soil processes were studied. The results showed that the relative agronomic efficiency compared with MinP decreased in the order: manure ≥fish sludge ≥wood ash ≥meat bone meal. The solubility of inorganic P in secondary resources was the main driver for P uptake by barley (*Hordeum vulgare*). The effects of secondary resources on physicochemical and microbial soil processes were of little overall importance. Application of organic carbon with manure resulted in microbial P immobilization and decreased uptake by barley of P derived from the soil. On both soils, P uptake by barley was best explained by a positive linear relationship with the H_2_O + NaHCO_3_-soluble inorganic P fraction in fertilizers or by a linear negative relationship with the HCl-soluble inorganic P fraction in fertilizers.

## Introduction

Minable rock phosphate is a finite resource. However, industrialized agroecosystems are today far from managing phosphorus (P) efficiently and Europe’s food production is largely dependent on imports of mined rock phosphate (1). The greatest reductions in P imports could be achieved by replacing mineral fertilizer with recycled P from secondary resources (2). In food systems, the accumulated P content in secondary resources is often of the same order of magnitude as that in mineral fertilizer, as shown for Europe (3). In Norway, the total amount of P in secondary resources (27,700 Mg P year^−1^) actually greatly exceeds the amount of P applied to soil with mineral fertilizer (8400 Mg P year^−1^) and the amount of P removed by crops (11,000 Mg P year^−1^) (4). The Norwegian secondary resources containing the largest amounts of P are manure (11,000 Mg P year^−1^), fish excrement, and feed losses from salmon and trout farming in open cages in fjords (fish sludge, 9000 Mg P year^−1^), meat bone meal (2100 Mg P year^−1^), and sewage (3100 Mg P year^−1^) (4). Wood ash, a residue from bioenergy plants and industrial timber production, also contains considerable amounts of secondary P (800 Mg P year^−1^) (5).

The P recycling potential of secondary resources is determined by, among other parameters, the solubility of the P species they contain (6, 7). Phosphorus in secondary resources is generally present as a complex mixture of inorganic P species, predominantly calcium (Ca) phosphates with differing solubility but also amorphous aluminum (Al)- or iron (Fe)-bound P, while organic P usually represents a small fraction (8). The P fertilization effects of secondary resources can be considerably affected by the pH in the target soil (7), as the solubility of Ca phosphates decreases with increasing soil pH, whereas the solubility of Al-/Fe-bound P decreases with decreasing soil pH (9). The best method for predicting the P fertilization effects of secondary resources also depends on the pH in the target soil (10). In a previous bioassay with ryegrass (*Lolium multiflorum*) grown in a sand–peat substrate containing little available P, the apparent P use efficiency of nine different secondary resources with predominantly Ca-bound P was best explained by a positive relationship with H_2_O-soluble inorganic P in acid soil and a negative relationship with HCl-soluble inorganic P in a near-neutral soil (7).

Plant P uptake following secondary resource application can also be influenced by their effects on microbial or physicochemical processes in the soil. Many secondary resources contain organic matter, meaning that organic carbon (C) is applied to the soil when they are used as alternatives to mineral fertilizers (e.g., manure, fish sludge, and meat bone meal). Organic C application can trigger microbial activity, which may result in immobilization of soil P and fertilizer P (11, 12) and in microbes competing with plants for available P. Microbial activity can also increase P availability by affecting physicochemical processes. Low molecular weight organic acids excreted by microorganisms during the breakdown of organic C have been shown to reduce phosphate retention on soil particles (13). Furthermore, these acids can solubilize P by complexing metal cations such as Al, Fe, and Ca that associate with P in insoluble forms, or by decreasing soil pH (14). Other secondary resources contain inorganic C, for example, in the form of calcium carbonate (CaCO_3_) (e.g., wood ash), which neutralizes pH in acid soils. Moreover, the solubility of applied fertilizer P can affect physicochemical soil processes depending on equilibrium processes in the target soil, since increased phosphate concentration in the soil solution can in turn result in reduced phosphate release from the soil (15).

To date, the P fertilization effects of fish sludge, meat bone meal, and wood ash have only been studied by the difference method (7, 16–18). This method compares the P uptake by a plant fertilized with the secondary resource with the P uptake by a plant receiving no P fertilizer (NoP). The difference in P uptake between the two treatments is defined as the P fertilization effect of the secondary resource. The underlying assumption in the difference method is that unfertilized and fertilized plants take up the same amount of P from the soil, i.e., that the secondary resource does not affect soil P availability. However, the effects of secondary resources such as fish sludge, meat bone meal, and wood ash on microbial and physicochemical soil P processes are still poorly understood, and it is not known whether the difference method actually reflects the net P fertilization effects of these secondary resources. Understanding the effects of complex secondary resources on soil processes is therefore important for a holistic evaluation of their fertilization effects.

Using radioisotopes of P in growth and incubation experiments provides the possibility to study P processes in soil/plant systems. In growth experiments, labeling soil with radioisotopes of P before application of secondary resources is a way to differentiate P taken up by the plants deriving from the fertilizer and from the soil (19–21). In incubation experiments, isotopic dilution can be used to study the incorporation of fertilizer into different soil P pools (22) or to quantify the amount of isotopically exchangeable phosphate (*E*-value) as affected by fertilizer application [e.g., Ref. (15, 20)].

The aim of this study was to determine the main drivers of plant P uptake following secondary resource application. To this end, the P fertilization effects of dairy manure (manure), fish sludge, meat bone meal, and wood ash were compared with those of water-soluble mineral P fertilizer (MinP) in a pot experiment with barley (*Hordeum vulgare* var. Heder) by ^33^P labeling of a loam soil at two soil pH levels (unlimed and limed). In parallel, soil incubation experiments were conducted to study the effects of the secondary resources on physicochemical and microbial soil processes and to explore their importance for plant P uptake.

## Materials and Methods

### Secondary Resources

The secondary resources are described in Table 1, while Table 2 gives an overview of their selected chemical properties. The secondary resources were also analyzed for heavy metal concentrations by ICP-OES after digestion in concentrated nitric acid in an ultraclave (23) (results not shown). Based on their heavy metal concentrations and Norwegian regulations (24), all the secondary resources studied here were eligible for application as fertilizer to agricultural land.

**Table 1:** **Description of secondary resources and identified P characteristics (7)**.

Product	Description	Inorganic P characteristics
Manure	Dried slurry (feces and urine) of dairy cows collected from the barn at the Norwegian University of Life Sciences, Norway	Mainly readily available (H_2_O-soluble) and labile (NaHCO_3_-soluble) Pi without further speciation
Fish sludge	Collected from the on-land Åsen settefisk salmon hatchery, Norway. Fish are bred in closed cages until they are approximately 1 year old. Effluent containing feces and feed residues (mainly fish meal and soya) was mechanically filtered before the material was treated on-site in a reactor developed by the company Global Enviro	Stable Ca-bound P such as apatite, but also amorphous Ca-bound P
Meat bone meal	Commercial product originating from a slaughterhouse in Hamar, Norway, and merchandized by Norsk Protein AS. Slaughterhouse waste of category III according to EC (25), which was stabilized and sanitized at 133°C and 3.0 bar for 20 min	Stable Ca-bound P, mainly apatite
Wood ash	Bottom ash from a grate-fired boiler system at the Moelven Østerdalsbruket AS mill, Norway. Parent material was timber unsuitable for industrial use	Stable Ca–P, mainly calcium phosphate silicate, and amorphous P. May also contain Al-/Fe-bound P

**Table 2:** **Selected chemical properties of secondary resources**.

		Manure	Fish sludge	Meat bone meal	Wood ash
Dry matter^a^	g 100 g^−1^	5.8	95.0	96.2	99.6
Organic matter^b^	g 100 g^−1^ DM	81.6	87.6	66.6	0.0
pH^c^		7.0	5.4	6.2	13
P^d^	g kg^−1^ DM	6	21	54	17
Po^d^	% of P	24	14	2	n.d.
${\text{P}}_{{\text{H}}_{2}\text{O}}$^e^	% of P	42	19	4	n.d.
${\text{P}}_{{\text{NaHCO}}_{\text{3}}}$^e^	% of P	33	19	5	43
P_NaOH_^e^	% of P	4	12	3	n.d.
P_HCl_^e^	% of P	2	37	88	63
C^f^	g kg^−1^ DM	470	503	368	19
Organic C^g^	g kg^−1^ DM	393	375	266	0.1
Organic ${\text{C}}_{{\text{H}}_{2}\text{O}}$^h^	% of organic C	14	34	41	n.d.
N^i^	g kg^−1^ DM	53	71	86	n.d.
Nmin^j^	g kg^−1^ DM	22	2.6	5.0	n.d.
K^k^	g kg^−1^ DM	42	3	4	56
Mg^k^	g kg^−1^ DM	6	3	3	25
S^k^	g kg^−1^ DM	42	48	34	34
Ca^k^	g kg^−1^ DM	11	37	110	310
Al^k^	g kg^−1^ DM	0.4	0.3	0.2	19.1
Fe^k^	g kg^−1^ DM	1.3	0.7	0.5	7.6

*Po, organic P; Nmin, mineral N $\text{(}NO_{\text{3}}^{-}\;and\;NH_{4}^{\text{+}}\text{)}$; n.d., not detectable*.

*^a^Drying of the original samples at 105°C*.

*^b^Incineration of the original samples at 550°C*.

*^c^Measured on dried and sieved (<2 mm) samples in H_2_O in a solid:solution ratio of 1:2.5 (v/v)*.

*^d^By ignition method on dried and milled samples before extraction with 6M H_2_SO_4_ according to Møberg and Petersen (26). Colorimetric analysis according to Murphy and Riley (27)*.

*^e^Sequentially extracted Pi of 1 g dried and milled sample in 200 mL H_2_O for 1 h, 200 mL 0.5M NaHCO_3_, 0.1M NaOH, and 1M HCl for each 16 h. Colorimetric analysis according to Murphy and Riley (27)*.

*^f^C/N elemental analysis (Leco TruSpec CHN) on dried and milled samples*.

*^g^Analyzed on triplicate dried and milled samples after washing with 2M HCl solution using a Perkin Elmer 2400 CHN analyzer*.

*^h^Extraction of 1 g dried and sieved sample in 200 mL H_2_O for 2 h, analyzed on Shimadzu TOC-V CPN*.

*^i^Modified Kjeldahl method (28) analyzed on Leco TruSpec CHN. Analyzed on a liquid sample of manure*.

*^j^Analyzed on Konelab Aqua 60 analyzer after extraction with 2M KCl (29, 30). Analyzed on a liquid sample of manure*.

*^k^Analyzed by ICP-OES after digestion of dried and milled samples with concentrated nitric acid in an ultraclave (23)*.

### Experimental Soil

The experimental soil originated from plots in a long-term field experiment in Norway (59°39′48.0″N 10°45′40.8″E) that has received 0 kg P year^−1^ and 0 or 5 kg potassium (K) year^−1^ since 1966. It is classified as an Albeluvisol in the World Reference Base for Soil Resources (31) and contains 27% clay, 40% silt, and 33% sand. Selected chemical characteristics of the soil are presented in Table 3. This soil was chosen because of its low content of plant-available P [measured as ammonium lactate-extractable P (P-AL)], in order to avoid P fertilization effects being masked by soil P. Before the soil was sampled at the end of the growing season in November 2013, barley, wheat and oats were grown in rotation for 16 years, with the last year of grass production being in 1997. After harvest of the cereals, including the straw, the soil was usually plowed in autumn. During sampling, random soil cores were taken from the 0–20 cm horizon in the middle of the plots. The soil was air-dried before sieving at mesh width 5 mm. To study the effect of soil pH on P uptake following secondary resource application, one part of the soil was limed with 2 g CaCO_3_ kg^−1^ soil dry matter (DM). Then, both the unlimed and the limed soils were incubated in portions of 15 kg at 60% of water-holding capacity (WHC, 100% WHC = 447 g H_2_O kg^−1^ soil) for 2.5 months in the dark before drying at 40°C. After transportation to Switzerland, the soil was again sieved at mesh width of 5 mm, carefully rewetted in portions of 1 kg soil DM, and incubated at 40% of WHC for at least 3 weeks. The pre-incubation aimed at reaching constant microbial activity, in order to minimize a microbial boost during setup of the experiment. When the experiments were set up, soil pH (measured in H_2_O) was 5.3 and 6.2 in the unlimed and limed soil, respectively.

**Table 3:** **Chemical properties of the soil**.

Organic matter^a^ (%)	Total P^b^ (mg kg^**−**1^)	Po^b^ (mg kg^**−**1^)	P-AL^c^ (mg kg^**−**1^)	K-AL^c^ (mg kg^**−**1^)	Mg-AL^c^ (mg kg^**−**1^)	Ca-AL^c^ (g kg^**−**1^)	Ox-Fe^d^ (g kg^**−**1^)	Ox-Al^d^ (g kg^**−**1^)
4.5	1024	456	44	138	44	1.3–1.9	4.8	1.9

*Po, organic P*.

*^a^Incineration at 550°C*.

*^b^By ignition method after extraction with 6M H_2_SO_4_ according to Møberg and Petersen (26). Colorimetric analysis according to Murphy and Riley (27)*.

*^c^Extraction with 0.1M ammonium lactate and 0.4M acetic acid adjusted to pH 3.75 according to Egnér et al. (32), analyzed on ICP-OES*.

*^d^Extraction with 0.2M ammonium oxalate in oxalic acid according to van Reeuwijk (33), analyzed by ICP-OES*.

### Pot Experiment

The P fertilization effects of secondary resources were studied in a pot experiment using indirect labeling with ^33^P (21). Pre-incubated portions of 1 kg soil DM were mixed with carrier-free ^33^P-orthophosphate at a rate of 1.1 MBq kg^−1^ soil, which was added after dilution in H_2_O by 10 mL kg^−1^ soil. The soil was transferred into pots with sealed bottoms and again incubated at 16–18°C for 10 days to reach near-equilibrium conditions for the pools of plant-available ^31^P and ^33^P in the soil. Pots containing the same amount of unlabeled soil were also mixed and kept under the same conditions. The fertilization effects of manure, fish sludge, meat bone meal, and wood ash (all dried at 55°C and sieved at ≤2 mm) were compared with those of a treatment receiving NoP and a treatment receiving water-soluble mineral P [MinP, Ca(H_2_PO_4_)_2_⋅H_2_O in aqueous solution]. For the purposes of methodological control, the fertilization effect of MinP was also studied using direct labeling (MinPdir). MinPdir was produced by labeling Ca(H_2_PO_4_)_2_⋅H_2_O in aqueous solution with specific activity (SA) 40 kBq mg P^−1^ and applied corresponding to 1.2 MBq kg^−1^ soil. All fertilizers were applied based on a total P content equivalent to 30 mg P kg^−1^ and mixed into the whole soil volume. This P dose corresponded to 5.09 g manure kg^−1^ soil, 1.48 g fish sludge kg^−1^ soil, 0.57 g meat bone meal kg^−1^ soil, and 1.76 g wood ash kg^−1^ soil. To study the response of the soil to P fertilization, unlabeled MinP was also applied at rates of 15 and 45 mg P kg^−1^ soil. At the same time, all pots received a P-free nutrient solution containing 75 mg N [Ca(NO_3_)_2_⋅4H_2_O], 75 mg K (K_2_SO_4_), 15 mg magnesium (Mg; MgSO_4_⋅7H_2_O), 0.1 mg molybdenum (Mo; Na_2_MoO_4_⋅2H_2_O), 1 mg zinc (Zn; ZnSO_4_⋅7H_2_O), 1 mg Fe (Fe-chelate), 1 mg boron (B; H_3_BO_3_), 2 mg copper (Cu; CuSO_4_⋅5H_2_O), and 2 mg manganese (Mn; MnSO_4_⋅H_2_O) per kg soil. There were four replicates per treatment. Seven barley seeds (*H. vulgare*, var. Heder) were sown per pot and thinned out to five plants after germination. Seventeen days after setup of the experiment, when plants had developed three to four leaves, all pots were also given 75 mg N and 209 mg K as KNO_3_. All plants were watered with distilled water by weighing to 70% of WHC until germination, thereafter to 60% of WHC every 2 or 3 days, and daily toward the end of the experiment. Growing conditions in the greenhouse were set to 16 h photoperiod with artificial lights turning on when daylight <20 klx. Atmospheric humidity and mean temperature were set to 65% and 20°C during the day and 72% and 16°C at night. Pot positions were randomized three times a week. Forty-four days after setup of the experiment, when the first awns were visible [development stage varying between Zadoks 35 and 50 (34)], aboveground biomass was harvested by cutting the plants with scissors at 2 cm above the soil surface. Plant material was dried at 55°C for 48 h, DM production per pot was recorded and the plant material was milled in a Retsch ZM 200 mill (≤0.2 mm). For determination of P concentration in the plant tissue, 250 mg were incinerated at 550°C for 3 h and extracted with 3 mL concentrated, hot HNO_3_ [adapted according to (22)]. The P in the diluted filtrate (0.2 μm pore size) was determined colorimetrically according to Ohno and Zibilske (35). The P uptake per kg soil was computed by multiplying DM production by plant tissue P concentration. The ^33^P beta emissions in the labeling solutions and the extracts were measured in 1 mL sample after addition of 5 mL appropriate scintillation liquid (PerkinElmer Ultima Gold or PerkinElmer Ultima Gold AB) by liquid scintillation counting (TRI-CARB 2500 TR, liquid scintillation analyzer, Packard Instruments, Meriden, CT, USA) and corrected for radioactive decay back to the day when the soil was labeled. The N concentration in plant tissue was determined using a Thermo Electron FlashEA 1112 Automatic Elemental Analyser. Soil samples were taken in each pot and soil pH was measured in a solid-solution-ratio of 1:2.5 (v/v) in H_2_O after drying soil samples at 55°C and sieving at mesh width ≤2 mm.

### Seed P Experiment

An additional experiment was conducted to determine the contribution of barley seed P to P uptake in aboveground biomass in response to increasing fertilization rate when the indirect method was used (22, 36). Sand (0.7–1.2 mm) was washed in 2% HCl before thorough rinsing with distilled H_2_O. Afterward, the pH of the sand was 4.97 [solid-solution-ratio of 1:2.5 (v/v) in H_2_O]. Portions of 1 kg sand DM were then fertilized with 0, 7.5, 15, 22.5 or 30 mg P kg^−1^ sand. The P fertilizer [Ca(H_2_PO_4_)_2_⋅H_2_O in aqueous solution] was labeled with ^33^P. The pots received 720 kBq kg^−1^ sand with the fertilizer, i.e., the SA of the P fertilizer was 96, 48, 32, and 24 kBq mg^−1^ P, respectively. The same P-free nutrient solution as given in the pot experiment was used. There were four replicates per treatment. Seven barley seeds were sown per pot and thinned out to five plants after germination. With five barley seeds, 0.71 ± 0.08 mg P were applied per kilogram of soil, as determined by the average weight of five barley seeds (0.20 ± 0.02 g DM kg^−1^, *n* = 20) and P concentration (3.49 ± 0.04 mg P g^−1^ DM, determined by colorimetric analysis after microwave digestion in concentrated H_2_O_2_ and HNO_3_, *n* = 4). During the first 19 days, the plants were watered up to 130 g H_2_O kg^−1^ sand, after which the water ratio was increased to 220 g H_2_O kg^−1^ sand. Plants were harvested by cutting with scissors at 2 cm above the sand surface 50 days after setup of the experiment when the first awns were visible (Zadoks 35–49). Plant material was analyzed in the same way as described for the pot experiment. During the seed P experiment, any isotopic dilution of the ^33^P in the shoot was caused by seed P, since this was the only non-labeled source. Therefore, this experiment allowed the P contribution from the seed and that from the fertilizer to be distinguished.

### Calculations for Pot and Seed P Experiment

When labeled fertilizer was applied to the soil (direct method in pot study and seed P experiment), P derived from the fertilizer (Pdf fertilizer, mg P kg^−1^ soil) was calculated as

(1) $$\text{Pdf fertilizer}=\frac{{\text{SA}}_{\text{plant}}}{{\text{SA}}_{\text{fert}}}\times {\text{P uptake}}_{{\text{P}}^{+}}$$

where SA_plant_ (Bq mg^−1^ P) is the SA in the plant amended with the labeled fertilizer, SA_fert_ (Bq mg^−1^ P) is the SA in the fertilizer, and ${\text{P uptake}}_{{\text{P}}^{+}}$ (mg P kg^−1^ soil) is the amount of P taken up by the fertilized plant in aboveground biomass. In the seed P experiment, P derived from the seed (Pdf seed) was calculated as the difference between P uptake and Pdf fertilizer.

**Figure 1:**
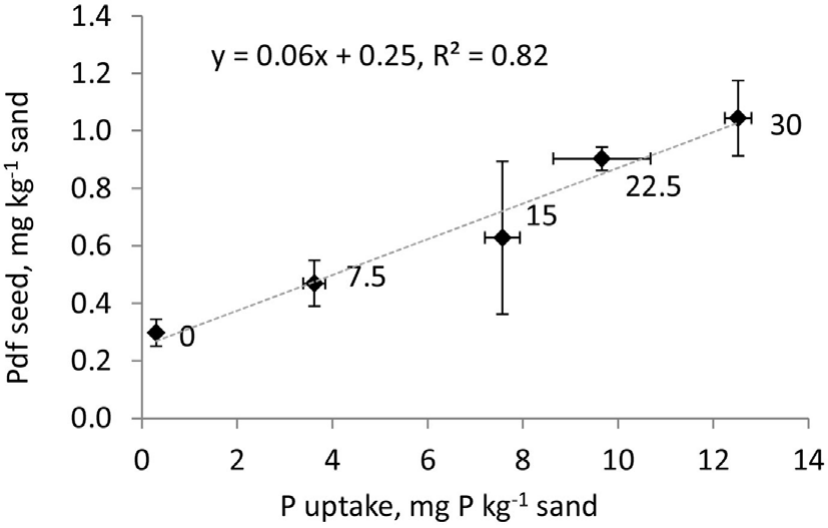
**Relationship between P uptake in aboveground biomass (mg P kg^−1^ sand) and P derived from the seed (Pdf seed, mg P kg^−1^ sand) for barley grown on sand and fertilized with increasing rates of MinP labeled with ^33^P (0, 7.5, 15, 22.5, and 30 mg P kg^−1^ sand as indicated next to the experimental points)**. Error bars represent SD of the four replicates.

When the pool of plant-available P in the soil was labeled before application of an unlabeled fertilizer (indirect method), Pdf fertilizer was calculated as

(2) $$\text{Pdf fertilizer}={\text{P uptake}}_{{\text{P}}^{+}}\;-{\text{Pdf soil}}_{{\text{P}}^{+}}\;-{\text{Pdf seed}}_{{\text{P}}^{+}}$$

where ${\text{Pdf soil}}_{{\text{P}}^{+}}$ is the amount of P derived from the soil (mg P kg^−1^ soil) in the fertilized plant, which was calculated as

(3) $${\text{Pdf soil}}_{{\text{P}}^{+}}=\frac{{\text{SA}}_{{\text{plant P}}^{+}}}{{\text{SA}}_{\text{plant NoP}}}\times \left({\text{P uptake}}_{{\text{P}}^{+}}\;-{\text{Pdf seed}}_{{\text{P}}^{+}}\right)$$

where ${\text{SA}}_{{\text{plant P}}^{+}}$ (Bq mg^−1^ P) is the SA in the fertilized plants, SA_plant NoP_ (Bq mg^−1^ P) is the average SA in the plants receiving NoP with P uptake corrected for Pdf seed, and ${\text{Pdf seed}}_{{\text{P}}^{+}}$ is P derived from the seed (mg P kg^−1^ soil) in the fertilized plants, which was calculated from the seed P experiment as follows:

(4) $${\text{Pdf seed}}_{{\text{P}}^{+}}=a\times {\text{P uptake}}_{{\text{P}}^{+}}\;+b$$

where *a* and *b* are the slope and intercept of the function presented in Figure 1.

Moreover, fertilizer recovery (%) was calculated as the ratio between Pdf fertilizer and the amount of P applied with the fertilizer (P fert, mg P kg^−1^):

(5) $$\text{Fertilizer recovery}=\frac{\text{Pdf fertilizer}}{\text{P fert}}\times 100$$

Relative agronomic efficiency (RAE, %) was calculated as the ratio between the fertilizer recovery of each secondary resource (P^+^) and the fertilizer recovery of MinP applied at the same rate (30 mg P kg^−1^ soil) as the secondary resource:

(6) $$\text{Relative agronomic efficiency}=\frac{{\text{Fertilizer recovery}}_{{\text{P}}^{+}}}{{\text{Fertilizer recovery}}_{\text{MinP}}}\times 100$$

### Incubation Experiment

#### Setup of the Incubation Experiment

In parallel, soil–fertilizer incubations were conducted to study physicochemical and microbial soil processes affected by the secondary resources in comparison with MinP and NoP on the unlimed and limed soil. Again, the soil was pre-incubated with distilled H_2_O at 40% of WHC to minimize a microbial boost during setup of the experiments. Three weeks after pre-incubation, aliquots of 1.2 kg soil were mixed with ^33^P corresponding to 5.2 MBq kg^−1^ soil that was added after dilution in H_2_O by 10 mL kg^−1^ soil for an incubation experiment with soil P labeling (indirect method). As for the pot experiment, the soil was kept in a controlled environment to reach near-equilibrium conditions for ^31^P and ^33^P for 10 days. A soil–fertilizer incubation experiment with no soil or fertilizer P labeling (termed unlabeled incubation) was also set up to determine isotopically exchangeable P as affected by P fertilizer (method described below). During the setup of both incubations, aliquots of 300 g soil were watered to 50% of WHC and mixed with fertilizer corresponding to 30 mg P kg^−1^ soil, but in contrast to the pot experiment, no P-free nutrient solution was added. Sub-aliquots of 100 g soil corresponding to three time points were placed in sealed plastic bags and kept in the dark under identical experimental conditions as in the pot experiment until analysis. There were four replicates per treatment. The effect of fertilizers on soil pH was studied after 7, 21, and 42 days in the incubation experiment with soil P labeling as described above.

#### P Concentration in Soil Solution and Isotopically Exchangeable P

The effects of fertilizers on the P concentration in the soil solution (*C*_P_, mg P L^−1^) and isotopically exchangeable P (*E*_1min_, in mg P kg^−1^) (37) were determined after 21 days in soil sampled in the unlabeled incubation experiment. The *E*_1min_ comprises the Pi in the soil solution and Pi adsorbed to soil particles, which is exchangeable within the first minute of isotopic exchange kinetics (21). Incubated soil samples were dried at 40°C before extraction of 10 g soil in 99 mL H_2_O by end-over-end shaking. Isotopic exchange kinetics analyses were carried out after 16 h of shaking based on the assumption that steady-state equilibrium was reached, i.e., that *C*_P_ was constant. The suspensions were then stirred at 300 rpm on a magnetic plate when 1 mL carrier-free ^33^P solution with a known amount of radioactivity was added to result in *R* = 600–900 Bq mL^−1^ in the sample. The soil:solution ratio was then 1:10. The suspensions were sampled with plastic syringes at 1, 4, 10, 30, 60, and 90 min after ^33^P addition (*t*). The remaining ^33^P in the filtrate [*r*(*t*), 0.2 μm pore size] was determined using scintillation counting as described above. After the last sampling, *C*_P_ was determined colorimetrically (35). The *E*_1min_ was calculated based on the assumption that *R* was evenly diluted with all inorganic P fractions having the same isotopic composition, according to

(7) $$E_{1\text{min}}=10\times C_{\text{P}}\times \frac{R}{r\left(1\right)}$$

(8) $$\frac{r\left(t\right)}{R}=m\times {\left[t+{\left(m\right)}^{\frac{1}{n}}\right]}^{-n}+\frac{10\times C_{\text{P}}}{\text{Pi}}$$

where Pi is the sum of Pi in the experimental soil (Total P minus Po; Table 3) and the fertilization rate 0 or 30 mg P kg^−1^. The isotopic dilution parameters *m* and *n* were calculated from a non-linear regression between *r*(*t*)/*R* and *t* before statistical refinement. The isotopic dilution parameter *m* is a measure of the remaining radioactivity in the solution after 1 min and *n* is a measure of how fast the radioactivity is disappearing from the solution.

#### Resin-Extractable P

The effects of fertilizers on resin-extractable P (Resin P) were studied after 7 and 21 days on soil sampled in the indirectly labeled incubation experiment. Moist samples equaling 2 g soil DM were extracted in 30 mL H_2_O upon horizontal shaking at 160 rec min^−1^ for 16 h with simultaneous adsorption to anion-exchange resin membranes (BDH 55164 2S, 6 cm × 2 cm) that had been shaken in advance twice in 0.5M NaHCO_3_ for 1 h. The P adsorbed to resin membranes was extracted by 0.1M NaCl/0.1M HCl. The P concentrations and radioactivity were determined as described above. The indirect labeling allowed estimation of the fraction of Resin P deriving from the fertilizer (Pdff Resin P, %) according to

(9) $$\text{Pdff Resin P}=\left(1-\frac{{\text{SA}}_{{\text{P}}^{+}}}{{\text{SA}}_{\text{NoP}}}\right)\times 100$$

where ${\text{SA}}_{{\text{P}}^{+}}$ (Bq mg^−1^ P) is the SA in the soil amended with fertilizer and SA_NoP_ (Bq mg^−1^ P) is the SA in the soil receiving NoP.

#### Microbial P

The effects of fertilizer on P in microbial biomass (Pmic) were estimated after 7 and 21 days in the indirectly labeled incubation experiment as the difference between extracted P with simultaneous adsorption to anion-exchange resin membranes from fumigated and non-fumigated soil samples (Resin P). For the fumigation extraction, moist soil equaling 2 g soil DM was extracted in 30 mL H_2_O with 1 mL hexanol for 16 h. As a methodological control, 2 g soil DM were extracted in 30 mL H_2_O using anion-exchange resin membranes after addition of a P spike of 10 μg P g^−1^ soil for 16 h. The test showed that released P was effectively sorbed to the anion-exchange resin membranes, and it was not necessary to correct microbial biomass P for sorption to soil of P released during the fumigation-extraction.

### Statistical Analysis

Equation 8 was adjusted using a non-linear procedure. Two-way ANOVA was applied to test the effect of the factors fertilizer treatment and pH level and their interaction on parameters studied in the pot and incubation experiment. Data sets were also analyzed using one-way ANOVA within the unlimed and the limed soil, respectively. Directly labeled treatments were excluded from the variance analyses and presented separately, including the SD of four replicates. Analyzed data were checked for normal distribution (normal quantile plots) and homogeneity of variance (residual versus fitted plots), and log transformed if indicated. For pair-wise comparisons, Tukey’s HSD test or *t*-tests were used at significance level α = 0.05. Moreover, simple linear regressions were run with selected parameters of the pot experiment as response variables and the parameters of the incubation experiment as explanatory variables, which were averaged over the four replicates. All statistical analyses were performed with JMP Pro 11.1.1 (38).

## Results

### P Concentration, Dry Matter Production, and Total P Uptake

There was a clear response of barley to P application on the experimental soil, as shown by linear increases in P uptake in aboveground biomass as a function MinP application rate (0, 15, 30, and 45 mg P kg^−1^ soil) on both the unlimed and limed soil. The slopes of the response curves for the two soils were not significantly different (*p* = 0.75), while the intercept was significantly higher on the limed than on the unlimed soil (*p* < 0.01) (see Supplementary Material). Phosphorus concentration in plant biomass ranged from 1.6 to 2.2 mg P g^−1^ DM. The P nutrition index for temperate grasses, calculated according to Liebisch et al. (39), clearly indicated P limitation in all fertilizer treatments (results not shown). Nitrogen concentrations (3.0–4.4 g 100 g^−1^ DM, results are not shown) were clearly above critical levels in temperate grasses as calculated according to Lemaire et al. (40), and observed differences between fertilizer treatments were therefore ascribed to P fertilization effects rather than N fertilization effects.

All secondary resources resulted in equally high P concentration as MinP on both soils except manure, which resulted in significantly lower P concentration than MinP on the limed soil. Aboveground DM production ranged from 3.5 to 5.2 g kg^−1^ soil and was equally high on the unlimed and limed soil (Table 4). None of the secondary resources increased DM compared with NoP on either soil, while MinP significantly increased DM compared with NoP on both soils. Phosphorus uptake in aboveground biomass ranged from 5.7 to 10.8 mg P kg^−1^ soil and was as result of slightly higher P concentration on the limed soil on average 0.5 mg P kg^−1^ soil greater on the limed than on the unlimed soil. All secondary resources resulted in significantly lower P uptake than MinP on both soils, except fish sludge, which resulted in equally large P uptake as MinP on the limed soil (Table 4).

**Table 4:** **Aboveground dry matter production (DM), P concentration and P uptake in aboveground biomass, P derived from fertilizer (Pdf fertilizer, %), fertilizer recovery (%), and relative agronomic efficiency (RAE, %) as an effect of different fertilizer treatments on unlimed and limed soil**.

Treatment	Dry matter (g DM kg^−1)^		P concentration (mg P g^−1^ DM)		P uptake (mg P kg^−1^ soil)		Pdf fertilizer (%)		Fertilizer recovery (%)		RAE (%)	
**Unlimed soil**
NoP	3.5	b	1.6	b	5.7	c						
MinP	5.2	a	1.9	a	10.2	a	42.4	a	14.4	a	100^a^	
Manure	4.1	b	1.7	ab	7.0	bc	40.2	ab	8.8	b	60.9	a
Fish sludge	4.0	b	1.9	a	7.5	b	29.0	bc	6.9	b	47.8	a
Meat bone meal	3.5	b	1.8	ab	6.4	bc	11.2	d	2.3	c	16.0	b
Wood ash	3.7	b	1.9	a	7.1	bc	23.6	cd	5.6	bc	38.7	ab
SEM	0.2		0.1		0.4		2.9		0.9		5.6	
HSD	0.8		0.3		1.6		12.8		4.0		23.4	
MinPdir	5.0 ± 0.3		2.1 ± 0.2		10.3 ± 0.6		36.9 ± 2.5		12.7 ± 0.6		n.d.	
**Limed soil**
NoP	3.5	b	1.8	ab	6.4	b						
MinP	5.0	a	2.2	a	10.8	a	45.1	a	16.3	a	100^a^	
Manure	4.6	ab	1.8	b	8.3	b	41.0	a	10.7	b	65.7	a
Fish sludge	4.5	ab	1.9	ab	8.7	ab	28.6	b	7.9	bc	48.6	ab
Meat bone meal^b^	3.7	b	1.9	ab	7.0	b	18.8	b	4.4	c	26.9	b
Wood ash	3.9	ab	1.9	ab	7.6	b	28.1	b	7.1	bc	43.8	ab
SEM	0.3		0.1		0.5		2.6		1.2		6.7	
HSD	1.1		0.4		2.4		11.3		5.1		28.6	
MinPdir	4.9 ± 0.4		2.3 ± 0.1		11.2 ± 1.0		33.5 ± 0.2		12.5 ± 1.1		n.d.	
**Two-way ANOVA, source of variation**
Treatment	***		***		***		****		***		***	
Soil	n.s.		*		**		n.s.		*		n.s.	
Treatment × soil	n.s.		n.s.		n.s.		n.s.		n.s.		n.s.	

*SEM, pooled SEM; HSD, Tukey’s honest significant difference at each pH level, where values followed by the same letter are not significantly different; n.s., not significant, n.d., not determined. For MinPdir mean ± SD of four replicates*.

*, **, and *** significant at *p* < 0.05, 0.01, and 0.001 probability level, respectively.

*^a^By definition set to 100%*.

*^b^Only three observations due to Pdf fertilizer <0 for one replicate*.

### P Uptake from Different Sources

**Figure 2:**
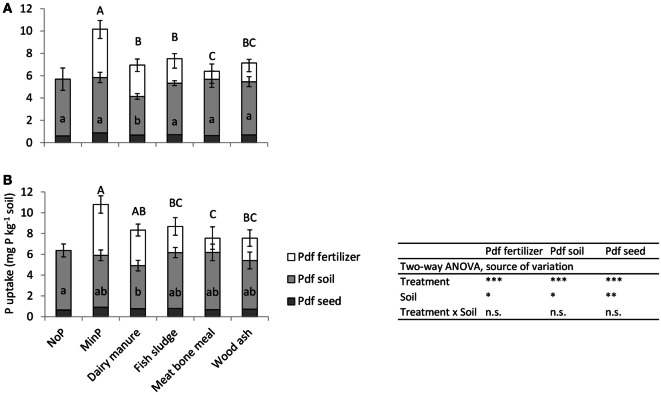
**Phosphorus derived from fertilizer (Pdf fertilizer), soil (Pdf soil), and seed (Pdf seed) in barley (mg P kg^−1^ soil) as an effect of the different fertilizer treatments on (A) unlimed soil and (B) limed soil**. Error bars represent the SD within each treatment. Letters indicate significant differences between treatments according to Tukey’s test (one-way ANOVA for each soil); uppercase letters refer to Pdf fertilizer and lowercase letters to Pdf soil. On the unlimed soil, data on Pdf soil were log transformed for the statistical analysis. On the limed soil, for Pdf fertilizer of meat bone meal, only three observations were considered. *, **, and *** significant at *p* < 0.05, 0.01, and 0.001 probability level, respectively.

Phosphorus derived from soil was the most important P source for barley plants with all fertilizer treatments on both soils (Figure 2). All treatments resulted in equally large Pdf soil, except manure, which resulted in significantly smaller Pdf soil than all other treatments on the unlimed soil and in smaller Pdf soil than NoP on the limed soil.

Phosphorus derived from fertilizer was significantly smaller after application of secondary resources than after MinP on both soils (Figure 2). Only manure resulted in equally large Pdf fertilizer as MinP on the limed soil. Fish sludge and wood ash resulted in equally large Pdf fertilizer as manure, while meat bone meal resulted in significantly smaller Pdf fertilizer than manure on both soils. The Pdf fertilizer accounted for 40–44% of P uptake in plants after application of MinP or manure, and in significantly smaller fractions after application of the other secondary resources compared with MinP (Table 4). Fertilizer recovery was 14–16% of applied MinP and was significantly lower after application of all secondary resources on both soils. The RAE decreased in the order manure ≥fish sludge ≥wood ash ≥meat bone meal on both soils, but only meat bone meal resulted in significantly lower RAE than manure. Fish sludge and wood ash resulted in equally high RAE as manure.

The MinP treatment resulted in significantly smaller Pdf soil than MinPdir, the average difference being 1 mg P kg^−1^, and in significantly larger Pdf fertilizer than MinPdir, the difference being 0.7 mg P kg^−1^ (two-sided *t*-tests over both soils, *n* = 8). The variability between replicates was also generally lower for MinPdir than for MinP and all other treatments.

The Pdf soil and Pdf fertilizer were corrected for Pdf seed as estimated from the relationship between P uptake and Pdf seed studied in the seed P experiment (Figure 1). In the seed P experiment, Pdf seed significantly increased with increasing P fertilization rate. The P uptake by the highest P fertilization rate (1.04 ± 0.15 mg P kg^−1^) in aboveground biomass was higher than the average amount of P applied with the seeds (estimated to be 0.71 ± 0.08 mg P pot^−1^). With *a* and *b* from Figure 1, 0.65–0.95 mg P pot^−1^ in aboveground biomass was estimated to derive from seeds, representing 8.6–10.7% of P uptake in the pot experiment. The ranking of Pdf soil and fertilizer among treatments did not change when not corrected for Pdf seed (see Supplementary Material), emphasizing the low importance of Pdf seed in the present study.

### Physicochemical and Microbial Soil Processes

On the unlimed soil, all fertilizers initially increased available P over NoP in the incubation experiment, as reflected by Resin P, measured 7 days after application (Table 5). Seven days after fertilizer application, Pdf fertilizer in the Resin P pool ranged from 25 to 38%, but there were no significant differences between treatments. Twenty-one days after application, Resin P was on average 4.6 mg P kg^−1^ soil lower than 7 days after fertilizer application (two-sided *t*-test over both time points, *n* = 48), and only MinP resulted in higher Resin P than NoP. The Pdf fertilizer in the Resin P pool ranged from 19 to 52%, again without significant differences between treatments. Twenty-one days after application, all secondary resources increased *C*_P_ over NoP and resulted in equally high *E*_1min_ as MinP.

**Table 5:** **pH measured in the pot experiment after harvest, *C*_P_ = P concentration in the soil solution, *m* and *n* = isotopic dilution parameters, *E*_1min_ = isotopically exchangeable P within 1 min, Resin P = resin-extractable P, Pdff Resin = P derived from the fertilizer in the resin-extractable P pool (%), and Pmic = microbial P 7 and 21 days after fertilizer application as an effect of different fertilizer treatments on unlimed and limed soil**.

Treatment	pH		C_P_^a^ Day 21 (mg P L^−1)^		*m* Day 21		*n* Day 21		*E*_1min_ Day 21 (mg P kg^−1)^		Resin P Day 7 (mg P kg^−1)^		Resin P^a^ Day 21 (mg P kg^−1)^		Pdff Resin Day 7 (%)		Pdff Resin Day 21 (%)		Pmic^d^ Day 7 (mg P kg^−1)^		Pmic Day 21 (mg P kg^−1)^	
**Unlimed soil**
NoP	5.51	bc	0.09	c	0.27	ab	0.40	a	3.3	b	12.5	b	10.1	b					2.8	n.s.	6.2	bc
MinP	5.49	c	0.15	a	0.32	a	0.38	b	4.8	a	21.6	a	25.2	a	38^b^	n.s.	52^b^	n.s.	3.7^b^	n.s.	3.6^b^	c
Manure	5.58	b	0.13	ab	0.29	ab	0.39	ab	4.6	a	23.7	a	13.9	b	32	n.s.	38	n.s.	6.4	n.s.	9.7	ab
Fish sludge	5.45	c	0.12	b	0.27	ab	0.39	ab	4.4	ab	19.1	a	16.6	ab	37^b^	n.s.	30	n.s.	5.8	n.s.	3.0	c
Meat bone meal	5.44	c	0.12	b	0.27	ab	0.39	ab	4.5	a	21.5	a	10.1	b	25	n.s.	19	n.s.			9.9	a
Wood ash	5.73	a	0.12	b	0.26	b	0.39	ab	4.4	ab	19.1	a	13.9	b	30	n.s.	40	n.s.	9.2	n.s.	5.0	c
SEM	0.02		0.01		0.01		0.00		0.3		1.1		2.2		7		7		1.6		0.8	
HSD	0.09		0.03		0.05		0.02		1.2		4.7		9.9		35		35		7.0		3.7	
**Limed soil**
NoP	6.25	bc	0.08	b	0.23	bc	0.37	a	3.5	c	10.3	c	11.8	b					9.1	bc	6.8	c
MinP	6.23	bcd	0.15	a	0.27	a	0.35	c	5.4	a	16.7	ab	23.4	a	43	n.s.	41	a	18.8	a	6.1	c
Manure	6.30	b	0.13	a	0.24	abc	0.35	bc	5.3	ab	18.8	a	16.9	ab	49	n.s.	40	a	14.7	ab	15.1	a
Fish sludge	6.17	cd	0.10	b	0.24	ab	0.37	a	4.2	bc	9.7	c	13.2	b	35	n.s.	24	ab	14.8	ab	11.9	ab
Meat bone meal	6.15	d	0.09	b	0.22	c	0.37	ab	4.2	bc	12.5	bc	14.2	b	29^c^	n.s.	12	b	7.0	c	6.7	c
Wood ash	6.45	a	0.14	a	0.22	bc	0.34	c	6.1	a	13.7	abc	15.5	b	40	n.s.	43^b^	a	13.1	abc	9.8	bc
SEM	0.02		0.01		0.01		0.00		0.3		1.2		1.7		6		5		1.5		0.9	
HSD	0.08		0.03		0.03		0.02		1.2		5.4		7.6		26		26		6.9		4.3	
**Two-way anova, source of variation**
Treatment	***		***		***		***		***		***		***		n.s.		***		*		***	
Soil	***		*		***		***		**		***		***		n.s.		n.s.		***		***	
Treatment × soil	n.s.		**		n.s.		**		**		*		**		n.s.		n.s.		*		***	

*HSD, Tukey’s honest significant difference at each pH level, where values followed by the same letter are not significantly different; n.s., not significant*.

*, **, and *** significant at *p* < 0.05, 0.01, and 0.001 probability level, respectively.

*^a^Two-way ANOVA based on log transformation*.

*^b^Only three observations*.

*^c^Only two observations*.

*^d^One-way ANOVA without meat bone meal on unlimed soil and two-way ANOVA without meat bone meal*.

On the limed soil, meat bone meal and fish sludge did not increase available P over NoP as reflected by *C*_P_, *E*_1min_, and Resin P at any time point. Wood ash did not increase Resin P over NoP at any time point on the limed soil, but resulted in equally high *C*_P_ and *E*_1min_ as MinP. Seven days after fertilizer application, there were no differences in Pdf fertilizer in the Resin P pool between fertilizer treatments, with values ranging from 29 to 49%. Twenty-one days after fertilizer application, meat bone meal was the only secondary resource that resulted in significantly lower Pdf fertilizer in the resin-extractable P pool (12%) than MinP (41%).

In the pot experiment, wood ash increased soil pH compared with NoP on both soils and meat bone meal resulted in significantly lower soil pH than NoP on the limed soil, while the other fertilizers had no significant effect on soil pH compared with NoP in the pot experiment (Table 5). In the incubation experiment, there were no differences in soil pH between the three time points, but soil pH was generally 0.4 and 0.2 pH units lower than in the pot experiment on the unlimed and limed soil, respectively. In the incubation experiment, wood ash resulted in significantly higher soil pH than NoP on both soils, and the effects of fertilizer treatments generally followed a similar pattern as in the pot experiment. The results are therefore not shown.

Phosphorus uptake in microbial biomass (Table 5) was of the same order of magnitude as P uptake in plants (Table 4). It was generally higher on the limed than on the unlimed soil, the average difference being 8.4 mg P kg^−1^ soil at 7 days and 3.1 mg P kg^−1^ soil at 21 days after fertilizer application. On the unlimed soil, there were no differences in Pmic between treatments, except an increase over NoP following meat bone meal application 21 days after fertilizer application. On the limed soil at 7 days after fertilizer application, only MinP had significantly increased Pmic over NoP. However, this effect was transient, as 21 days after fertilizer application Pmic of MinP was significantly lower than at 7 days after fertilizer application, and MinP and meat bone meal resulted in equally low Pmic as NoP. In contrast, manure and fish sludge had significantly increased Pmic over NoP.

### Drivers of P Uptake by Barley

**Figure 3:**
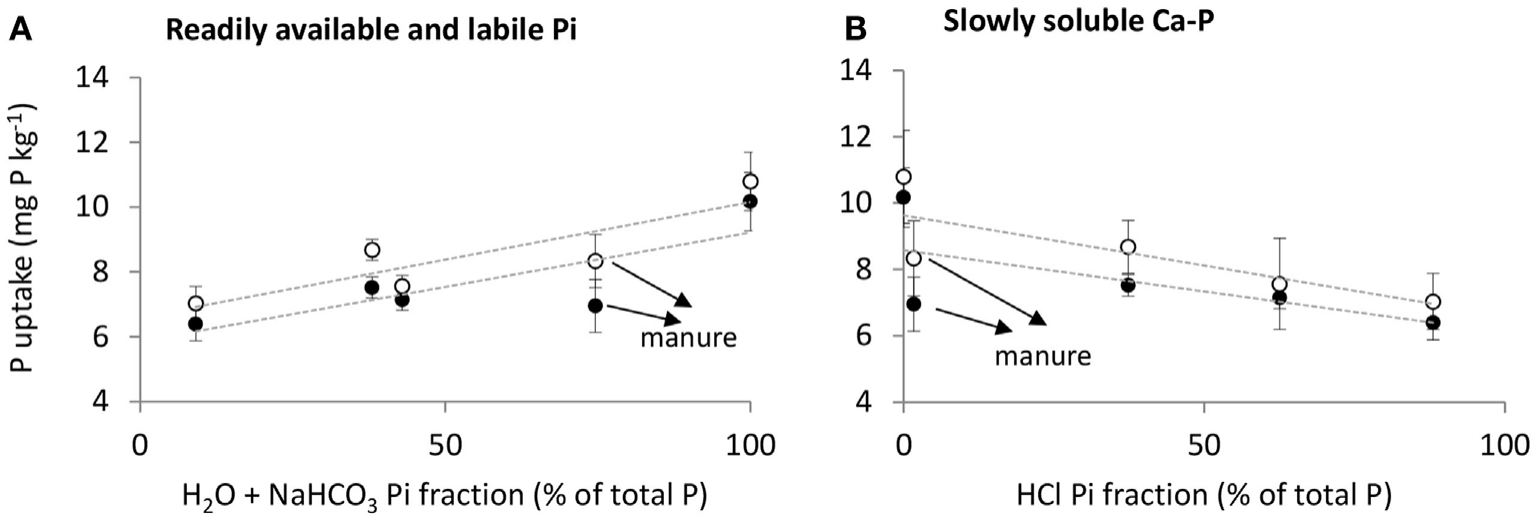
**Phosphorus uptake by barley (mg P kg^−1^ soil) as an effect of (A) H_2_O + NaHCO_3_-soluble inorganic P fraction and (B) HCl-soluble inorganic P fraction in fertilizers for both soils (∙ = unlimed soil and ∘ = limed soil)**. All P in MinP was assumed to be present in the H_2_O + NaHCO_3_ Pi fraction. Error bars indicate SD of Pdf fertilizer between replicates.

Phosphorus uptake by barley was best explained by the solubility of inorganic P in fertilizers, whereas additional effects of fertilizers on physicochemical and microbial soil processes were of little overall importance. This is shown by linear positive relationships between P uptake in barley and the H_2_O + NaHCO_3_-soluble inorganic P (Pi) fraction in fertilizers and linear negative relationships between P uptake and the HCl-soluble Pi fraction in fertilizers on both soils (Figure 3; Table 6). According to the sequential fractionation based on Hedley et al. (41), the H_2_O + NaHCO_3_-soluble Pi fraction is operationally defined as readily available and labile Pi, while the HCl-soluble Pi fraction is defined as the slowly soluble Ca–P fraction. Phosphorus uptake by barley could further be explained by *C*_P_, *m*, and Resin P measured 21 days after fertilizer application on both soils, which all represent measures for the solubility of fertilizer P applied to the soil. The Pmic measured 7 days after fertilizer application resulted in significant relationships with P uptake by barley. However, while the relationship was negative on the unlimed soil, it was positive on the limed soil. Soil pH was unable to explain the variation in P uptake by barley between fertilizer treatments.

**Table 6:** **Results of simple linear regression with Y = P uptake (mg P kg^−^^1^ soil) by barley plants on unlimed or limed soil without NoP, and *X* = explanatory variables, where fertilizer characteristics are sequentially extracted inorganic P (Pi) fractions in secondary P resources (Table 2), *C*_P_ = water-soluble P (mg P L^−^^1^), *m* and *n* = isotopic dilution parameters, *E*_1min_ = isotopically exchangeable P (mg P kg^−^^1^ soil), Resin P = resin-extractable P (mg P kg^−^^1^ soil), Pdff Resin P = P derived from the fertilizer in the resin-extractable P pool (%), and Pmic = microbial P (mg P kg^−^^1^ soil), measured, respectively, 7 and 21 days after fertilizer application**.

	Unlimed soil		Limed soil^a^	
	*R*^2^	*p*-value	*R*^2^	*p*-value
**Fertilizer characteristics**
H_2_O + NaHCO_3_-soluble Pi	0.55	***	0.47	***
HCl fraction	0.35	**	0.41	**
**Physicochemical soil processes**
*C*_P_	0.58	***	0.25	*
*m*	0.53	***	0.60	***
*n*	0.83^c^	***	0.05	n.s.
*E*_1min_	0.38	**	0.03	n.s.
Resin P, 7 days	0.00	n.s.	0.08	n.s.
Pdff resin P, 7 days	0.55	***	0.15	n.s.
Resin P, 21 days	0.84	***	0.44	**
Pdff resin P, 21 days	0.58	***	0.14	n.s.
Soil pH (pot experiment)	0.02	n.s.	0.02	n.s.
**Microbial soil processes**
P mic, 7 days	0.51^b,c^	**	0.50	***
P mic, 21 days	0.35	**	0.03	n.s.

*, **, and *** significant at *p* < 0.05, 0.01, and 0.001 probability level, respectively.

*n.s., not significant*.

*^a^Only three replicates of meat bone meal*.

*^b^Without meat bone meal*.

*^c^Negative relationship*.

## Discussion

### Effects of Inorganic P Species in Secondary Resources on P Uptake by Barley

The P uptake by barley following secondary resource application was mainly affected by the solubility of the inorganic P species the resource contained. The indirect effects of the secondary resources studied on P uptake through their influences on physicochemical and microbial soil processes were generally of less importance.

Poor P uptake following meat bone meal application can be explained by its large fraction of Ca-bound P such as hydroxyapatite and chlorfluorapatite (7) with low solubility, especially in soils with pH >6.5 (42). Similar results have been reported by Ylivainio et al. (18) and Brod et al. (7) after application of meat bone meal to ryegrass.

Fish sludge was the secondary resource that tended to result in the highest P uptake, probably because a considerable P fraction in fish sludge is readily available and labile, i.e., soluble in H_2_O and NaHCO_3_ (Table 2). However, fish sludge also contains apatite (7), which can explain why neither meat bone meal nor fish sludge increased *C*_P_, *E*_1min_, and Resin P over NoP in the limed soil of the incubation experiment.

Wood ash resulted in equally high P uptake as the other secondary resources, as expected from its large fraction of labile Pi, in addition to slowly soluble Ca–P [mainly (Ca_2_(SiO_4_))_6_Ca_3_(PO_4_)_2_] (7). The P uptake following wood ash application was equally high on both soils, but on the limed soil of the incubation experiment wood ash surprisingly increased *C*_P_ and *E*_1min_ to the same level as MinP. Similarly, Brod et al. (7) found increasing P fertilization effects of the same wood ash with increasing soil pH and attributed this effect to the likely presence of NaHCO_3_-soluble P adsorbed to Al-/Fe-(hydr)oxides. Wood ash was the secondary resource with the largest NaHCO_3_-soluble Pi fraction among all products studied. Phosphorus adsorbed to Al-/Fe-(hydr)oxides is characterized by increasing solubility with increasing soil pH (9). However, increased *C*_P_ and *E*_1min_ values after wood ash application on the limed soil could also be a methodological artifact if the magnetic stirrer mechanically destroyed (Ca_2_(SiO_4_))_6_Ca_3_(PO_4_)_2_, thereby solubilizing phosphate, while the same stable Ca–P was already solubilized in the unlimed soil. Similarly, Sinaj et al. (43) found that silicato-calcium phosphate present in Thomas slag quickly solubilized after application to an acidic soil with pH 6.2. A methodological artifact during determination of *E*_1min_ would explain why increased wood ash P solubility on the limed compared with the unlimed soil was not reflected by increased Pdf fertilizer in the pot experiment or elevated Resin P on the limed soil.

Manure resulted in lower P uptake by barley than MinP, even though 75% of P in manure was present as readily available and labile Pi (Table 2). Phosphorus uptake following manure application tended to be lower than expected from the linear regression lines with the H_2_O + NaHCO_3_-soluble or HCl-soluble Pi fractions as explanatory variables (Figure 3). Oberson et al. (44) also reported lower P fertilization effects of cow feces than di-ammonium phosphate after a pot experiment using indirect labeling and soils with different fertilization histories.

### Effects of Microbial Soil Processes on P Uptake by Barley

Our results indicate that organic C applied with manure may have resulted in microbial immobilization of soluble P, since Pdf soil (mg P kg^−1^) in barley was significantly lower after manure application than after NoP on both soils (Figure 2). This is also in agreement with manure increasing Pmic, compared with MinP, on both soils after 21 days (Table 5). Oberson et al. (44) reported microbial P immobilization following cow feces application to soils with different fertilization histories, and Bünemann et al. (45) describe increases in Pmic as a result of glucose addition during an incubation experiment with a P-deficient tropical soil. On the limed soil in the present study, total P uptake by barley was lower for the manure than the MinP treatment, but Pdf fertilizer (mg P kg^−1^) after manure application was equally high as after MinP. Therefore, the difference method would have underestimated the fertilization effect of manure because of microbial immobilization of soluble P in this soil and thereby a lower contribution from soil P.

Even though organic C also was applied with fish sludge and meat bone meal, microbial P immobilization seems not to have been a major competitor to barley plants in these cases, and Pdf soil (mg P kg^−1^) was equally high as after MinP. At the same fertilization rate of P, only 557 and 152 mg organic C kg^−1^ were applied with fish sludge and meat bone meal, compared with 2008 mg organic C kg^−1^ with manure (Table 2). In the incubation experiment, however, there were still signs of P immobilization, indicated as increased Pmic over NoP 21 days after application of fish sludge on the limed and meat bone meal on the unlimed soil (Table 5). This can be explained by a larger fraction of C in fish sludge and meat bone meal being soluble in H_2_O than the C in manure (Table 2). Accordingly, Bünemann et al. (46) point out a strong impact of C quality on microbial P immobilization. Still, due to too few products included in this study, we cannot specify the quantity and quality of organic C in secondary resources at which P uptake by plants might be negatively affected by activated microbial activity. Furthermore, it remains unknown whether the effect of secondary resources on microbial soil processes is also negligible for plant P uptake on soils with high microbial activity, because in the present study Pmic in both soils was overall rather low (47).

### Effects of Physicochemical Soil Processes on P Uptake by Barley

Phosphorus uptake by barley was higher on the limed than on the unlimed soil (Figure 2), probably because growing conditions were better overall. Barley is known to be sensitive to low soil pH [e.g., Ref. (48)], which is often associated with high concentrations of soluble Al. However, higher P uptake on the limed than on the unlimed soil could also be due to higher P availability, as supported by higher P concentration in plants receiving NoP (two-sided *t*-test, *n* = 8) (Table 4). On the unlimed soil, more P was probably adsorbed to Al-/Fe-(hydr)oxides because of increased positive surface charges at lower soil pH. The increased importance of P adsorption on the unlimed soil was also indicated by a decrease in Resin P over time, which was not observed in the limed soil (Table 5).

Wood ash caused a significant increase in soil pH on both soils as result of the liming effect of CaCO_3_. Increasing effects of wood ash on soil pH are well known [e.g., Ref. (49)]. However, the effect of wood ash on pH was far too small to significantly influence P availability in the soil (Table 6). Equally high Pdf soil (mg P kg^−1^) after wood ash application and NoP showed that soil P availability was not affected by the pH-increasing effect of wood ash within the pH range in the present experiment. With wood ash, only 0.1 g CaCO_3_ kg^−1^ soil was applied [8 wt% CaCO_3_ in wood ash according to Brod et al. (7)], in comparison with 2 g CaCO_3_ kg^−1^ soil applied to the limed soil.

### Methodological Considerations

Conducting the isotope dilution technique allowed us to conclude that the difference method would have led to similar results for P fertilization effects in this case, because P uptake by barley after secondary resource application was overall little affected by their influence on physicochemical and microbial soil processes. The underlying assumption in the difference method that fertilized and unfertilized treatments take up the same P amount from the soil would only have been violated after manure application due to microbial immobilization. Therefore, the results are also in agreement with those of a previous experiment in which a sand–peat mixture was used as a model soil (7), even though natural soil processes could not be studied. Thus, the results of the present study indicate that the difference method is reliable for secondary resources with low ratios of OM to P. However, these results should be confirmed with different soil types and extended with several secondary resources with a wide range of organic C content compared with P.

The indirect labeling method is based on the assumptions that plant-available soil P is homogeneously labeled and that dilution of the radioisotope is only due to the unlabeled fertilizer. The internal control treatment MinPdir resulted in significantly higher Pdf soil and lower Pdf fertilizer than MinP according to a comparison of the two treatments over both soils. The difference between the directly and indirectly labeled mineral control treatment suggests that unlabeled soil P contributed to the dilution of the SA in plants in the MinP treatment, e.g., *via* the mineralization of organic or microbial P (44). This means that fertilization effects might have been slightly overestimated when the indirect method was used. In fact, significantly increased Pmic over NoP after MinP application on the limed soil after 7 days indicates modified microbial activity also in the mineral control treatment, even though no organic C was applied with MinP. In that case, soil microbes were probably stimulated as a methodological artifact by introducing oxygen when mixing the experimental soil, as suggested by Oberson et al. (50) and Bünemann et al. (51). Twenty-one days after fertilizer application, the microbial boost could no longer be observed.

Another methodological inconsistency was observed in the seed P experiment, where our results incorrectly indicated that the amount of P derived from the seed was larger than the amount applied with seed. It is possible that the acid-washed sand still contained P, some P added with the two removed seeds had leaked into the soil, or P applied with the seed was underestimated because the seeds sown per pot were not weighed. In addition, large variation between the replicates of Pdf seed (Figure 1) and relatively low transfer from the seed compared with total P uptake might have contributed to the inconsistency. However, the actual reasons could not be identified. An alternative to the approach used here is to estimate Pdf seed by comparing the P content in the seeds at seedling stage and the remaining P content in the seeds at harvest, as suggested by Achat et al. (52). Several studies have pointed out the importance of correcting for seed P contribution when the indirect method is applied [e.g., Ref. (22, 36, 52)] to avoid overestimation of P derived from the unlabeled fertilizer. Here, the fraction of Pdf seed in total P uptake was small in comparison with, e.g., results reported by Achat et al. (52), who used ryegrass and fescue as the experimental crop and suggested that 15–50% of P uptake at the first cut was Pdf seed. The difference between the different indirectly labeled treatments was also small and the relative fertilization effect of the secondary resources was not affected by Pdf seed.

## Conclusion

This study explored the effects of secondary resources on physicochemical and microbial soil P processes and their importance for plant P uptake at two soil pH levels in the same arable soil. The main driver for P uptake was found to be the solubility of inorganic P species contained in the secondary resources, while indirect effects on P availability *via* influences on physicochemical and microbial soil processes were of little overall importance. This implies that P uptake following secondary resource application can be sufficiently predicted by intrinsic chemical P characteristics. The P uptake by barley was indeed best explained by a linear positive relationship with the H_2_O + NaHCO_3_-soluble inorganic P fraction and a linear negative relationship with the HCl-soluble inorganic P fraction in fertilizers on both unlimed and limed soil. Organic C resulted in microbial immobilization of labile P and decreased uptake in barley of P derived from the soil only after manure application. In this arable soil with rather low microbial biomass P, immobilization of P in microbial biomass could not challenge barley plants as the main P sink after application of fish sludge and meat bone meal. Further studies are needed to identify the critical organic C content in secondary resources at which microbial P processes influence plant P uptake. The significant increase in soil pH as result of wood ash application had no effect on P uptake by barley plants within the pH range in this study.

## Author Contributions

EB and AO designed the experiment; EB conducted the experiment; and EB analyzed the data with the help of AO and wrote the paper. All authors contributed to the interpretation of the results, and read and approved the manuscript.

## Conflict of Interest Statement

The authors declare that the research was conducted in the absence of any commercial or financial relationships that could be construed as a potential conflict of interest.

## Abbreviations

P fert, P applied with the fertilizer; P uptake, P taken up by plant in aboveground biomass; Pdf fertilizer, P derived from the fertilizer; Pdf seed, P derived from the seed; Pdf soil, P derived from the soil; Pdff ResinP, Resin P deriving from the fertilizer; Pi, inorganic P; RAE, relative agronomic efficiency; SA, specific activity.
