# Supplementary material for: Drivers of Phosphorus Uptake by Barley Following Secondary Resource Application
Source: Front Nutr. 2016 May 12;3:12. doi: 10.3389/fnut.2016.00012 (PMC4867682; doi:10.3389/fnut.2016.00012)
Supplement: Supplementary file 1 [file data_sheet_1.docx]

Supplementary material

Drivers of phosphorus uptake by barley following secondary resource application

Eva Brod 1,2*, Anne Falk Øgaard 1, Tore Krogstad 2, Trond Knapp Haraldsen 1, Emmanuel Frossard 3, Astrid Oberson 3

^1^ NIBIO, Norwegian Institute of Bioeconomy Research, P.O. Box 115, 1431 Ås, Norway

^2^ Norwegian University of Life Sciences, Department of Environmental Sciences, P.O. Box 5003, 1432, Ås, Norway

^3^ Institute for Agricultural Sciences, ETH Zurich, Eschikon 33, 8315 Linday, Switzerland

*** Correspondence:** eva.brod@nibio.no

**Appendix A: Response curves**

**Figure S1. P uptake by barley (mg P kg^-1^ soil) as an effect of P fertilization rate (0, 15, 30, 45 mg P kg^-1^ soil) on unlimed and limed soil. Error bars represent standard deviation of the four replicates.**

**Appendix B: Seed P correction**

B.1. No Pdf seed correction

To study the importance of seed P correction for the results obtained, Pdf soil and Pdf fertilizer as an effect of fertilizer treatment and soil were calculated and analysed without any seed P correction (Table S1). Comparing Pdf soil and Pdf fertilizer, the ranking between fertilizer treatments was not changed. This shows low importance of Pdf seed in the present study.

B.2. Pdf seed correction according to Oberson *et al.* (2010)

An alternative approach to estimating Pdf seed, based on the difference between proportional Pdf fertilizer after MinP and MinPdir application (Oberson *et al.*, 2010), was also used for each soil. Here, Pdf seed was assumed to be equal for all treatments on the respective soil and independent of P uptake. The proportional Pdf fertilizer after MinP and MinPdir was assumed to be identical. Phosphorus derived from the seed does not affect Pdf fertilizer determined using the direct method, because in MinPdir the fertilizer is labelled.

Pdf fertilizer_MinP_ (mg P kg^-1^) after MinP application without seed P correction was calculated as:

${Pdf fertiliz\mathrm{er}}_{\mathrm{MinP}}=(1-\frac{\mathrm{SA}_{plant MinP}}{\mathrm{SA}_{plant NoP}} )\times{P uptake}_{\mathrm{MinP}}$ (1)

where SA_plant MinP_ (Bq mg^-1^ P) is the specific activity in the plants fertilized with MinP, SA_plant NoP_ (Bq mg^-1^ P) is the average specific activity in the plants receiving no P fertilizer and P uptake (mg P kg^-1^ soil) is the amount of P taken up by the plants fertilized with MinP in aboveground biomass.

Pdf fertilizer_MinP_ (mg P kg^-1^) after MinP application with seed P correction was calculated as:

${Pdf fertiliz\mathrm{er}}_{\mathrm{MinP}}-Pdf seed=\frac{\mathrm{SA}_{plant MinPdir}}{\mathrm{SA}_{\mathrm{fert}}} \times{P uptake}_{\mathrm{MinP}}$ (2)

where SA_plant MinPdir_ (Bq mg^-1^ P) is the specific activity in the plants fertilized with MinPdir and SA_fert_ (Bq mg^-1^ P) is the specific activity in the MinPdir fertilizer.

Pdf seed (mg P kg^-1^ soil) after MinP application was hence calculated as:

$Pdf seed= \left[ \left( 1-\frac{\mathrm{SA}_{plant MinP}}{\mathrm{SA}_{plant NoP}} \right)- \frac{\mathrm{SA}_{plant MinPdir}}{\mathrm{SA}_{\mathrm{fert}}} \right]\times{P uptake}_{\mathrm{MinP}}$ (3)

With this approach, Pdf seed was again estimated to be larger than the amount of P applied with the seeds (0.71±0.08 mg P kg^-1^) on both soils. This confirms our assumption that the acid-washed sand still contained P, some P added with the two removed seeds had leaked to the soil, or more P might have been applied with the seeds than was determined. Pdf seed was estimated to be 0.85±0.59 mg P kg^-1^ on the unlimed soil and 1.63±0.42 mg P kg^-1^ on the unlimed soil (Table 1). Here, Pdf seed accounted for 8-15% of total P uptake on the unlimed soil and for 15-25% of total P uptake on the limed soil.

With this estimation approach, the complete difference in Pdf fertilizer between MinPdir and MinP is ascribed to Pdf seed. However, the difference in Pdf fertilizer between MinPdir and MinP could also be explained by unlabelled soil P contributing to the dilution of the specific activity in the plants, e.g. via mineralization of organic or microbial P during the indirect method (Oberson *et al.*, 2010). We assumed greater importance of microbial activity on the limed soil than on the unlimed soil, which could explain the larger Pdf seed on the limed compared with the unlimed soil. With this approach, there was no effect of soil pH on Pdf soil or Pdf fertilizer.

**Table S1. Comparison of P derived from soil (Pdf soil) and fertilizer (Pdf fertilizer) without Pdf seed correction and with Pdf seed correction according to Oberson *et al.* (2010)**

|  | No Pdf seed correction | | | | | Pdf seed correction according to Oberson *et al.* (2010) | | | | |
| --- | --- | --- | --- | --- | --- | --- | --- | --- | --- | --- |
| Treatment | Pdf seed | Pdf soil | | Pdf fertilizer | | Pdf seed | Pdf soil | | Pdf fertilizer | |
| Unlimed soil |  |  | |  | |  |  | |  | |
| NoP | 0 | 5.7 | a |  |  | 0.9 | 4.8 | a |  |  |
| MinP | 0 | 5.6 | a | 4.6 | a | 0.9 | 5.6 | a | 3.8 | a |
| Dairy manure | 0 | 3.9 | b | 3.1 | b | 0.9 | 3.3 | b | 2.8 | ab |
| Fish sludge | 0 | 5.2 | ab | 2.3 | b | 0.9 | 4.4 | ab | 2.3 | b |
| Meat bone meal | 0 | 5.6 | a | 0.8 | c | 0.9 | 4.7 | a | 0.8 | c |
| Wood ash | 0 | 5.3 | a | 1.8 | bc | 0.9 | 4.5 | ab | 1.8 | bc |
| SEM |  | 0.3 |  | 0.3 |  |  | 0.3 |  | 0.2 |  |
| HSD |  | 1.4 |  | 1.4 |  |  | 1.4 |  | 1.1 |  |
| MinPdir | 0 | 6.5±0.6 | | 3.8±0.2 | | 0.9 | 5.7±0.4 | | 3.8±0.2 | |
| Limed soil |  |  |  |  |  |  |  |  |  |  |
| NoP | 0 | 6.4 | a |  |  | 1.6 | 4.7 | ab |  |  |
| MinP | 0 | 5.6 | ab | 5.2 | a | 1.6 | 5.6 | a | 3.6 | a |
| Dairy manure | 0 | 4.6 | b | 3.7 | ab | 1.6 | 3.4 | b | 3.3 | a |
| Fish sludge | 0 | 6.0 | ab | 2.7 | bc | 1.6 | 4.4 | ab | 2.6 | ab |
| Meat bone meal | 0 | 6.1 | ab | 1.5^a^ | c | 1.6 | 4.5 | ab | 1.4^a^ | b |
| Wood ash | 0 | 5.2 | ab | 2.3 | bc | 1.6 | 3.9 | b | 2.1 | ab |
| SEM |  | 0.4 |  | 0.4 |  |  | 0.3 |  | 0.4 |  |
| HSD |  | 1.6 |  | 1.7 |  |  | 1.5 |  | 1.6 |  |
| MinPdir | 0 | 7.5±0.7 | | 3.8±0.3 | | 1.6 | 5.9±0.7 | | 3.8±0.3 | |
| Two-way ANOVA, source of variation | | | | | | | | | | |
| Treatment |  | *** | | *** | |  | *** | | *** | |
| Soil |  | * | | * | |  | n.s. | | n.s. | |
| Treatment x soil |  | n.s. | | n.s. | |  | n.s. | | n.s. | |

SEM = pooled standard error of the mean and HSD = Tukey’s honest significant difference at each pH level, where values followed by the same letter are not significantly different. For MinPdir mean ± standard deviation of 4 replicates. *, **, *** significant at p < 0.05, 0.01 and 0.001 probability level, n.s. = not significant

^a^Only three observations due to Pdf fertilizer < 0 for replicate 4.
